# Opioids and Gabapentinoids Utilisation and Their Related-Mortality Trends in the United Kingdom Primary Care Setting, 2010–2019: A Cross-National, Population-Based Comparison Study

**DOI:** 10.3389/fphar.2021.732345

**Published:** 2021-09-14

**Authors:** Amanj Kurdi

**Affiliations:** ^1^ Strathclyde Institute of Pharmacy and Biomedical Science, University of Strathclyde, Glasgow, United Kingdom; ^2^ Department of Pharmacology and Toxicology, College of Pharmacy, Hawler Medical University, Erbil, Iraq; ^3^ Division of Public Health Pharmacy and Management, School of Pharmacy, Sefako Makgatho Health Sciences University, Pretoria, South Africa

**Keywords:** opioids, gabapentinoids, utilisation trends, cross-national comparison study, prescription cost analysis

## Abstract

**Background:** There is growing concern over the increasing utilisation trends of opioids and gabapentinoids across but there is lack of data assessing and comparing the utilisation trends across the four United Kingdom countries. We assessed/compared opioids and gabapentinoids utilisation trends across the four United Kingdom countries then evaluated the correlation between their utilisation with related mortality.

**Methods:** This repeated cross-national study used Prescription Cost Analysis (PCA) datasets (2010–2019). Opioids and gabapentinoids utilisation were measured using number of items dispensed/1,000 inhabitants and defined daily doses (DDDs)/1,000 inhabitant/day. Number of Opioids and gabapentinoids-related mortality were extracted from the United Kingdom Office for National Statistics (2010–2018). Data were analysed using descriptive statistics including linear trend analysis; correlation between the Opioids and gabapentinoids utilisation and their related mortality using Pearson correlation coefficient.

**Results:** The results illustrated an overall significant increasing trend in the utilisation of opioids (12.5–14%) and gabapentinoids (205–207%) with substantial variations among the four United Kingdom countries. For opioids, Scotland had the highest level of number of items dispensed/1,000 inhabitant (156.6% higher compared to the lowest level in England), whereas in terms of DDD/1,000 inhabitant/day, NI had the highest level. Utilisation trends increased significantly across the four countries ranging from 7.7% in Scotland to 20.5% in NI (*p* < 0.001). Similarly, for gabapentinoids, there were significant increasing trends ranging from 126.5 to 114.9% in NI to 285.8–299.6% in Wales (*p* < 0.001) for number of items/1,000 inhabitants and DDD/1,000 inhabitant/day, respectively. Although the utilisation trends levelled off after 2016, this was not translated into comparable reduction in opioids and gabapentinoids-related mortality as the latter continued to increase with the highest level in Scotland (3.5 times more deaths in 2018 compared to England- 280.1 vs. 79.3 deaths/million inhabitants). There were significant moderate-strong positive correlations between opioids and gabapentinoids utilisation trends and their related mortality.

**Conclusion:** The utilisation trends of opioids and gabapentinoids have increased significantly with substantial variations among the four United Kingdom countries. This coincided with significant increase in their related mortality. Our findings support the call for immediate actions including radical changes in official United Kingdom policies on drug use and effective strategies to promote best clinical practice in opioids and gabapentinoids prescribing.

## Introduction

Opioids are analgesics that have been widely used as the standard care for treating severe acute and chronic pain including cancer pain (Rosenblum et al., 2008). However, there a growing concerns over their increasing use in treating chronic non-cancer pain (CNCP) (Noble et al., 2010; Bedson et al., 2016). This is despite the lack of evidence to support their effectiveness in treating chronic pain beyond short-term moderate benefit (Kalso et al., 2004). Opioids use can be potentially dangerous and associated with adverse effects and harm to patients. Adverse effects can include abuse, addiction, dependence, diversion and increased mortality, particularly when used long-term and at higher doses (Chou et al., 2015; Els et al., 2017). This is reflected by a progressive rise in opioid-related harm and mortality in many developed countries associated with the marked increasing trend of opioid use for patients with CNCP (Jauncey et al., 2005; Centers for Disease Control and Prevention (CDC), 2011; Gladstone et al., 2016). Similarly, in the United Kingdom there has been a marked and progressive increase in opioids prescribing over the last decade (The National Institute for Health and Care Excellence, 2019). In England, there was a 34% increase in opioids prescribing between 1998 and 2016 (Curtis et al., 2019). In Scotland, opioid prescribing increased by 250% between 2002 and 2015 (Torrance et al., 2018). This increasing in opioids use coincided with a rising number of opioid-related deaths in England and Wales; out of the 4,359 reported drug-related deaths in 2018, 51% were related to opioids use (Office for National Statistics, 2019a). Similarly, in Scotland, out of the 1,187 drug-related deaths (almost doubled compared to 2008), opioids were involved in 86% (1,021 deaths) of them (National Records of Scotland, 2019a).

Similar issues in terms of progressive increasing prescribing trends and mortality has been observed with gabapentinoids (gabapentin and pregabalin). These two medicines were initially indicated to treat epilepsy. However, they have increasingly been prescribed for pain management particularly in treating neuropathic pain as second line treatment (National Institute for Health Care and Excellence, 2020). However, they have been increasingly prescribed off-label to treat other pain conditions despite the lack of conclusive effectiveness evidence (Shanthanna et al., 2017; Peckham et al., 2018). Gabapentinoids also have the potential for dependence, abuse and misuse. This is because they are sought as a recreational drug due to their reinforcing subjective effects, such as euphoria, sedation and dissociation (Schifano et al., 2011), with sufficient evidence indicating that gabapentinoids can be used for non-medical indications (Evoy et al., 2017). Similar to opioids, there has been a substantial increase in gabapentinoids prescribing and mortality in the United Kingdom. For instance, there was a 393 and 900% increase in gabapentin and pregabalin prescribing, respectively, between 2007 and 2017 in England (The National Institute for Health and Care Excellence, 2019). There has also been a marked increase in gabapentinoids-related mortality in Scotland from 2 deaths in 2008 to 367 deaths in 2018 constituting 31% of all reported drug-related mortality in 2018 (National Records of Scotland, 2019a). This increasing use of pregabalin in the United Kingdom may help to explain why the Company were very keen to protect their business for neuropathic pain when their patent for epilepsy expired. The company achieved this aim by threatening legal action for any physician who prescribed pregabalin by its INN rather than by its brand name (Godman et al., 2015).

Although previous studies have studied the prescribing trends of opioids in the United Kingdom, these studies are either out-dated (i.e., limited up to 2014 or 2015) (Cartagena Farias et al., 2017; Mordecai et al., 2018), and/or limited to individual United Kingdom component countries such as only England (Mordecai et al., 2018; Curtis et al., 2019). To the best of our knowledge no previous studies have been undertaken evaluating opioids and gabapentinoids prescribing trends across the four United Kingdom countries or assessing their utilisation trends in association with related mortality. Evaluating gabapentinoids prescribing trends is of particular importance as they could have been prescribed as replacement for opioids due to their opioids-sparing effects in the likely mistaken belief about their less likelihood of being misused or causing dependence (Savelloni et al., 2017; The National Institute for Health and Care Excellence, 2017). This is of particular concern given government’s attempts to regulate and control opioids consumption such as the reclassification of tramadol in 2014 from a Schedule 4 to Schedule 3 drug (Advisory Council on the Misuse of Drugs, 2013). Given the recognised role of comparative prescribing data in monitoring and improving clinical practice and current lack of knowledge (Ivers et al., 2012), the objective of this study was to evaluate and compare prescribing trends for opioids and gabapentinoids across the four United Kingdom countries (England, Scotland, Wales, and Northern Ireland). Subsequently, assess the strength of association (correlation) of their utilisation trends with opioids and gabapentinoids-related mortality using the most available recent national data.

## Methods

### Study Design, Data Source and Study Subjects

This study was an observational, retrospective repeated cross-national study using the publicly available Prescription Cost Analysis (PCA) datasets of England (NHS Digital, 2019), Scotland (ISD Scotland, 2019), Wales (Prescribing Services NWSSP, 2019) and Northern Ireland (Health and Social Care Business Services Organisation, 2019) (NI) from 2010 to 2019. PCA datasets contain aggregated-level information on all prescribed and dispensed prescriptions in the United Kingdom primary care setting. This includes the medicine’s name, strength, quantity, cost and formulation. We extracted dispending data of gabapentinoids (gabapentin and pregabalin), and all opioids preparations that are indicated for pain relief stratified into strong opioids (tramadol, morphine, fentanyl, oxycodone, hydromorphone, pethidine, tapentadol, alfentanil, diamorphine, pentazocine, methadone and buprenorphine) and week opioids (codeine, dihydrocodeine, meptazinol and dextropropoxyphene) based on the content of the British National Formulary (BNF) (Royal Pharmaceutical Society, 2019). Opioids preparations that are mostly indicated for opioids substitution therapy included in section 4.10 of the BNF (including some methadone and buprenorphine preparations) were excluded as these are unlikely to be used for pain management. Data on opioids and gabapentinoids-related mortality were extracted from the United Kingdom Office for National Statistics (Office for National Statistics, 2019b) and the National Records of Scotland (National Records of Scotland, 2019b) from 2010 to 2018 (the most up-to-date data available at the time of the study) whereby data on annual number of deaths and reasons for deaths are recorded; subsequently, we extracted all death records for which opioids and/or gabapentinoids were the recorded reason of death. Ethical approval was not required as this study used publicly available datasets in the United Kingdom. This is similar to other studies we have conducted using publicly available datasets in the United Kingdom (Godman et al., 2018; Godman et al., 2019).

### Study Outcomes

The study outcomes were the opioids and gabapentinoids utilisation trends and mortality related to their use. The utilisation trends were measured using two utilisation metrics: annual number of dispensed items/1,000 inhabitants and annual defined daily dose (DDD)/1,000 inhabitants/day, stratified by strong and weak opioids.

For the former, we extracted the total number of items dispensed in each year for each of the 4 countries during the study period from PCA divided by the total population size and multiplied by 1,000 in order to obtain a standard denominator to ensure that the observed trends are not just an artefact of either the variation in population size among the four countries or the annual alteration in population size over time. For the population size for each country, we used mid-year population size estimates for each corresponding year and country extracted from the United Kingdom Office of National Statistics (Office for National Statistics, 2019c). DDDs are the assumed average maintenance dose per day for a drug used for its main indication for an adult (World Health Organization Collaborating Centre for Drug Statistics and Methodology, 2020). DDDs are often presented as DDDs/1,000 inhabitants/day as an internationally recognised utilisation metric that accounts for population sizes for comparative purposes within different regions of a country or across countries (World Health Organization, 2003; Godman et al., 2018), We calculated DDDs/1,000 inhabitants/day by summing the annual total dispensed quantity (in mgs) for each included opioids and gabapentinoids (extracted from PCA) and adjusted by their corresponding assigned DDD values (World Health Organization Collaborating Centre for Drug Statistics and Methodology, 2020). Subsequently divided by mid-year population size, multiplied by 1,000 and divided by 365 (Chen et al., 2019); for combination products, we divided the annual dispensed quantity (e.g., tables) by the their assigned DDD values based on their number of daily unit doses as per WHO guidance (World Health Organization Collaborating Centre for Drug Statistics and Methodology, 2020). The annual number of opioids and gabapentinoids-related mortality were extracted and presented as per 1 million inhabitants to accounts for the variations in the population size among the four United Kingdom countries.

### Data Analysis

Descriptive statistics were used to describe the utilisation trends overtime. Changes in utilisation trends during the study period were presented as absolute and relative percentage changes. Liner regression was used to perform a trend analysis overtime to obtain the average annual changes in utilisation overtime. The correlation (strength of association) between the opioids and gabapentinoids utilisation and related mortality trends was assessed using Pearson coefficient (Sedgwick, 2012) and presented as a correlation coefficient (range from 1- to 1) as a mean of hypothesis generation; similar approach has been used in other studies using ecological, aggregated dataset (Ganmaa et al., 2002) We did not include in the analysis the time points where the various relevant opioids and gabapentinoids related-polices were introduced in the United Kingdom because assessing the impact of these policies was not the focus of our current study; besides, the impact of some of these policies such as the re-classification of tramadol (Advisory Council on the Misuse of Drugs, 2013) has already been assessed and published in a previous study (Chen et al., 2018).

### Patient Involvement

Patients were neither involved in the development of the research question, the study outcomes nor in the design, implementation of the study, interpretation, or writing up the results.

## Results

### Utilisation Trends for Opioids

**FIGURE 1:**
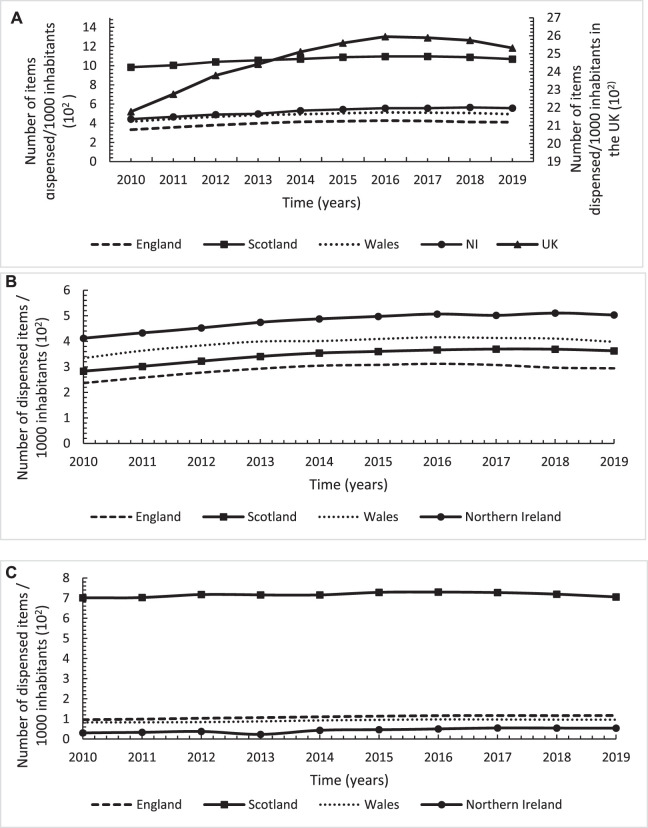
Annual utilisation trends in number of dispensed items/1,000 inhabitants for opioids across England, Scotland, Wales and Northern Ireland over the study period from 2010 to 2019; **(A)**: Total opioids; **(B)**: Strong opioids; **(C)**: Weak opioids.

Overall, there was a 14% (*n* = 354) increase in the number of dispensed items/1,000 inhabitants from 2,177 in 2010 to 2,532 in 2019 (Figure 1), with a significant average annual increase of 41.5 dispensed items/1,000 inhabitants (*p* < 0.001), driven mainly by the high utilisation trend in Scotland. However, the trend was increasing up until 2016 when it started and continued to decline by 2.5% (*n* = 43) from 2,589 in 2016 to 2,532 in 2019. Across the four countries, there was substantial variations with the highest utilisation trend in Scotland and the lowest in England, with Scotland having 159.6% (*n* = 657) higher number of dispensed items/1,000 inhabitants in 2019 compared to England (Figure 1). Furthermore, although all four countries showed significant increasing trends over time, with the highest increase in NI followed by Scotland, the increasing trend levelled off after 2016 and continued to decline up until the end of the study period (Figure 1; Table 1). Upon stratifying the utilisation by strong and weak opioids, similar utilisation trends could be seen but with NI having the highest utilisation of strong opioids, followed by Scotland, but the lowest utilisation of weak opioids with the highest being in Scotland (more than 7 times higher than NI) (Figure 1; Table 1).

**TABLE 1:** Absolute, relative, and average annual changes in the utilisation trends of opioids across England, Scotland, Wales and Northern Ireland over the study period from 2010 to 2019.

	England	Scotland	Wales	Northern Ireland
Annual number of dispensed items/1,000 inhabitants				
Total opioids				
Absolute change (relative % change)	78.8 (18.4%)	84.2 (7.7%)	77.1 (15%)	114.3 (20.5%)
Annual average change	8.6^a^	10.7^a^	8.6^a^	13.6^a^
Strong opioids				
Absolute change (relative % change)	57.7 (18.5%)	79.6 (21.5%)	64 (15.4%)	91 (17.9%)
Annual average change	6.1^a^	9.1^a^	6.8^a^	13.6^a^
Weak opioids				
Absolute change (relative % change)	21.1 (18.1%)	4.6 (0.6%)	13.0 (13.3%)	23.3 (46.4%)
Annual average change	2.5^a^	1.6	1.8^a^	2.3^a^
Annual number of DDD/1,000 inhabitants/day				
Total opioids				
Absolute change (relative % change)	3.0 (12.2%)	2.7 (9.2%)	1.1 (6.1%)	4.8 (12.5%)
Annual average change	0.3^a^	0.5^a^	0.1	0.8^a^
Strong opioids				
Absolute change (relative % change)	1.9 (11.0%)	1.0 (4.7%)	1.0 (7.9%)	4.2 (12.0%)
Annual average change	0.2	0.3	0.1	0.7^a^
Weak opioids				
Absolute change (relative % change)	1.7 (15.4%)	1.7 (20.5%)	0.1 (1.3%)	0.5 (17.2%)
Annual average change	0.1^a^	0.2^a^	0.03	0.1^a^

a (Note): Indicates *p*-value < 0.05, obtained from the liner regression analysis; DDD: defined daily dose.

**FIGURE 2:**
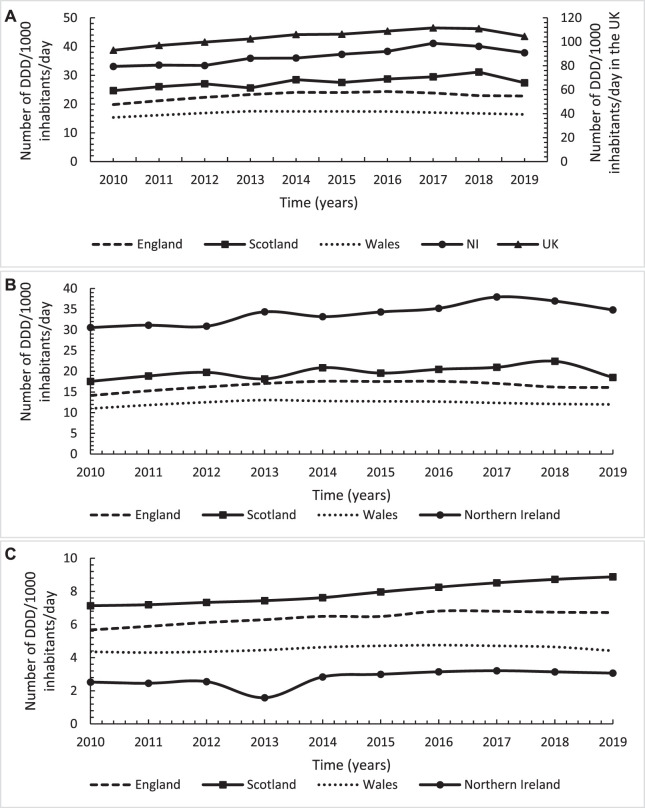
Annual utilisation trends in number of defined daily doses/1,000 inhabitants/day for opioids across England, Scotland, Wales and Northern Ireland over the study period from 2010 to 2019; **(A)**: Total opioids; **(B)**: Strong opioids; **(C)**: Weak opioids.

In terms of DDD/1,000 inhabitants/day, similar utilisation trend was observed with an overall increasing trend of 12.5% (*n* = 12) from 93 in 2010 to 105 in 2019 (Figure 2), with significant average annual increase of 1.7 DDD/1,000 inhabitants/day (*p* < 0.001). However, the increasing utilisation trend levelled off after 2016 as the trend declined by 17% (*n* = 4) from 109 in 2016 to 105 in 2019. In contrast to the number of dispensed items/1,000 inhabitants, NI had the highest utilisation trend regarding the number of DDDs/1,000 inhabitants/day followed by Scotland with the lowest in Wales (Figure 2). Likewise, despite the significant increasing trends over time across the four countries, with the highest increase in NI followed by Scotland, the increasing trends levelled off after 2016 (Figure 2; Table 1). In terms of stratifying the utilisation by strong and weak opioids, the observed utilisation trends for DDDs/1,000 inhabitants/day was comparable to the utilisation trends for number of dispensed items/1,000 inhabitants (Figure 2; Table 1).

### Utilisation Trends for Gabapentinoids

**FIGURE 3:**
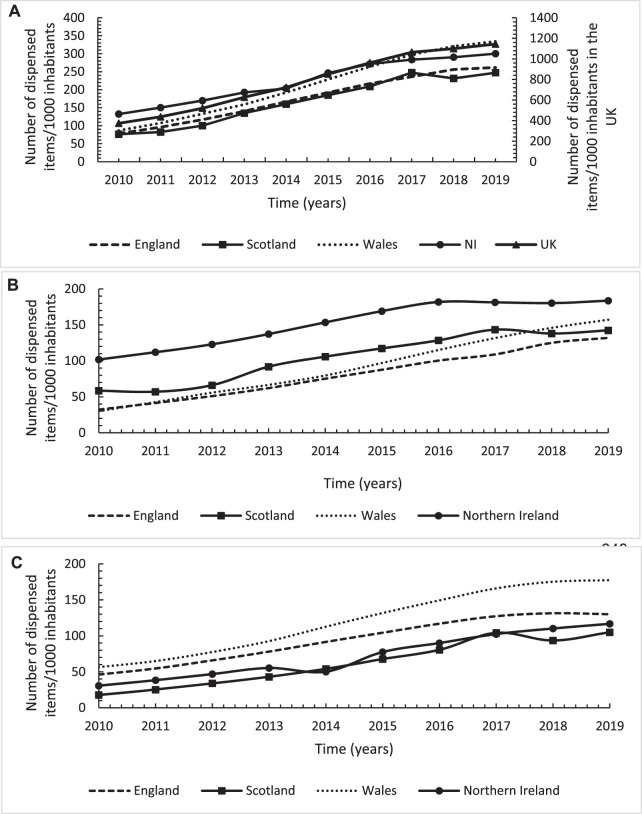
Annual utilisation trends in number of dispensed items/1,000 inhabitants for gabapentinoids across England, Scotland, Wales and Northern Ireland over the study period from 2010 to 2019; **(A)**: Total gabapentinoids; **(B)**: Pregabalin; **(C)**: Gabapentin.

Overall, there was a steady increase of 205.8% (*n* = 770) in the number of total gabapentinoids dispensed items/1,000 inhabitants in the United Kingdom from 374 in 2010 to 1,144 in 2019 (Figure 3). Whilst, there was a significant annual average increase of 93.5 dispensed items/1,000 inhabitants (*p* < 0.001) over the study period, the increasing trend was at a lower rate from 2016 to 2019 compared to 2010–2016 [39.5 dispensed items/1,000 inhabitants/year (*p* = 0.04) vs. 100 (*p* < 0.001), respectively). There was a wide variation across the four United Kingdom countries with the highest increase in Wales but a comparable increase across England, Scotland and NI (Figure 3; Table 2). At the start of the study period in 2010, NI had the highest gabapentinoids utilisation but by 2019, Wales had superseded NI as the country with the highest utilisation adjusted for the population size (Figure 3). In terms of the individual gabapentinoids, pregabalin accounted for the majority of the gabapentinoids use in NI and Scotland with the highest utilisation level in NI, even though the highest increasing rate was in Wales (Figure 3; Table 2). For gabapentin, Wales accounted for the highest utilisation level and increasing rate followed by England (Figure 3; Table 2).

**TABLE 2:** Absolute, relative, and average annual changes in the utilisation trends of gabapentinoids across England, Scotland, Wales and Northern Ireland over the study period from 2010 to 2019.

	England	Scotland	Wales	Northern Ireland
Annual number of dispensed items/1,000 inhabitants				
Total gabapentinoids				
Absolute change (relative % change)	183.6 (233.8%)	170.9 (223.6%)	247.8 (285.8%)	167.7 (126.5%)
Annual average change	22^a^	21.6^a^	29.7^a^	20.2^a^
Pregabalin				
Absolute change (relative % change)	99.7 (309.1%)	83.8 (143%)	127 (421%)	81.7 (80.2%)
Annual average change	11.5^a^	11.1^a^	14.6^a^	10^a^
Gabapentin				
Absolute change (relative % change)	83.9 (181.3%)	87.1 (488.6%)	120.8 (213.75)	86 (279.9%)
Annual average change	10.5^a^	10.5^a^	15.1^a^	10.2^a^
Annual number of DDD/1,000 inhabitants/day				
Total gabapentinoids				
Absolute change (relative % change)	9.1 (218.3%)	14.1 (270.6%)	12.8 (299.6%)	9.7 (114.9%)
Annual average change	1.1^a^	1.8^a^	1.5^a^	1.2^a^
Pregabalin				
Absolute change (relative % change)	5.5 (263%)	8.7 (546.9%)	7.2 (388.2%)	5.9 (86.2%)
Annual average change	0.6^a^	1.1^a^	0.8^a^	0.8^a^
Gabapentin				
Absolute change (relative % change)	3.6 (173.6%)	5.5 (150.4%)	5.6 (231.7%)	3.8 (233.6%)
Annual average change	0.5^a^	0.7^a^	0.7^a^	0.5^a^

a (Note): Indicates *p*-value < 0.05, obtained from the liner regression analysis; DDD: defined daily dose.

**FIGURE 4:**
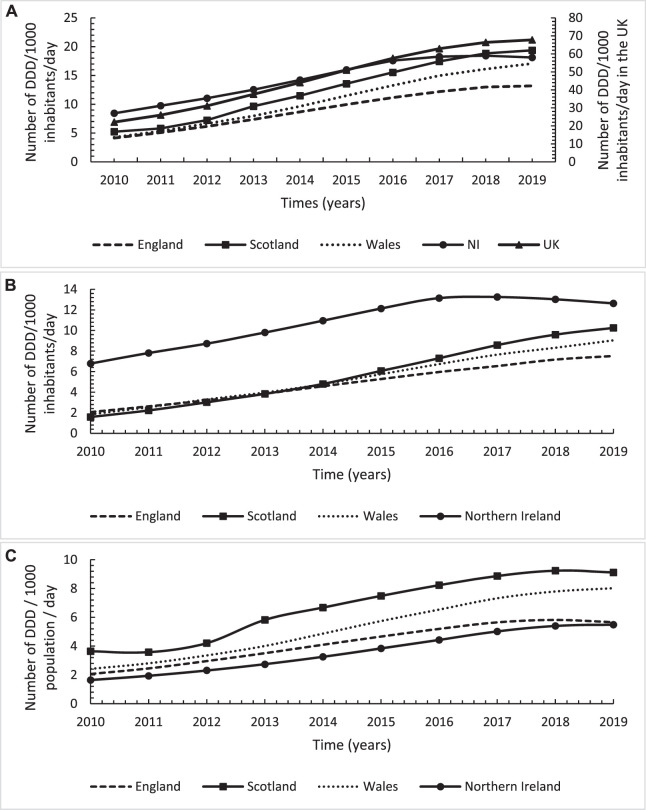
Annual utilisation trends in number of defined daily dose/1,000 inhabitants for gabapentinoids across England, Scotland, Wales and Northern Ireland over the study period from 2010 to 2019; **(A)**: Total gabapentinoids; **(B)**: Pregabalin; **(C)**: Gabapentin.

Similarly, the utilisation in terms of the number of DDDs/1,000 inhabitants/days showed a significant overall increasing trend of gabapentinoids of 207% (*n* = 46) over the study period in the United Kingdom (Figure 4). However, unlike the number of items, Scotland had the highest utilisation level and increasing rate (Figure 4; Table 2). Furthermore, NI has the highest utilisation level for pregabalin followed by Scotland but with the latter having the highest increasing rate overall (Figure 4; Table 2). For gabapentin, Scotland had the highest utilisation level followed by Wales with both countries having a similar increasing rate overall.

### Opioids and Gabapentinoids-Related Mortality

**FIGURE 5:**
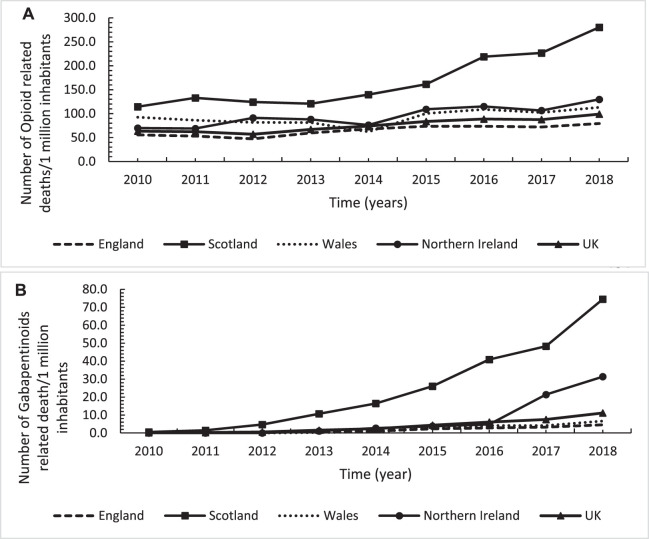
Annual trends in number of deaths per one million inhabitants for opioids **(A)** and gabapentinoids **(B)** across the United Kingdom, England, Scotland, Wales and Northern Ireland over the study period from 2010 to 2018.

Overall, there was a 56.6% (*n* = 35.8) increasing trend in the number of opioids-relating mortality per one million inhabitants in the United Kingdom, with a significant annual increasing rate of five opioids-relating mortality per one million inhabitants (Figure 5). The trends level of opioids-relating mortality for all the four United Kingdom countries, except England, were above the average United Kingdom level but with the highest level observed in Scotland. In Scotland, there was a 144.8% (*n* = 165.7) increase over the study period with a significant annual increasing rate of 19.6 (*p* < 0.001) deaths per one million inhabitants (Figure 5; Table 3). Overall, Scotland had 3.5 times more deaths in 2018 compared to the lowest observed rate in England (280.1 vs. 79.3, respectively). Similarly, there was a substantial overall rise of gabapentinoids-related deaths by eight times over the study period in the United Kingdom and again with the highest increasing level and rate in Scotland (12 times increase with a significant annual increasing rate of 8.7 death per one million inhabitants), in particular after 2015 for Scotland and 2016 for NI (Figure 5; Table 3). Scotland had 16 times more deaths in 2018 compared to the lowest observed rate in England (74.5 vs. 4.6, respectively).

**TABLE 3:** Absolute, relative, and average annual changes in the trends of opioids and gabapentinoids-related mortality and its association with their utilisation patterns across England, Scotland, Wales and Northern Ireland over the study period from 2010 to 2018.

	England	Scotland	Wales	Northern Ireland
Opioids-related mortality				
Absolute change (relative % change)	23.6 (42.4%)	165.7 (144.8%)	20.6 (25.3%)	59.9 (68%)
Annual average change	3.6^a^	19.6^a^	3.4	7.0^a^
Pearson correlation coefficient	0.84^a^	0.78^a^	0.42	0.87^a^
Gabapentinoids -related mortality				
Absolute change (relative % change)	4.5 (4,734%)	73.9 (12,963%)	6.7 (2,062%)	31.4 (2,868%)
Annual average change	0.6^a^	8.7^a^	1.3^a^	6.0^a^
Pearson correlation coefficient	0.97^a^	0.91^a^	0.94^a^	0.77^a^

a (Note): Indicates *p*-value < 0.05.

### Associations of Opioids and Gabapentinoids-Related Mortality With Their Utilisation Trends Overtime

**FIGURE 6:**
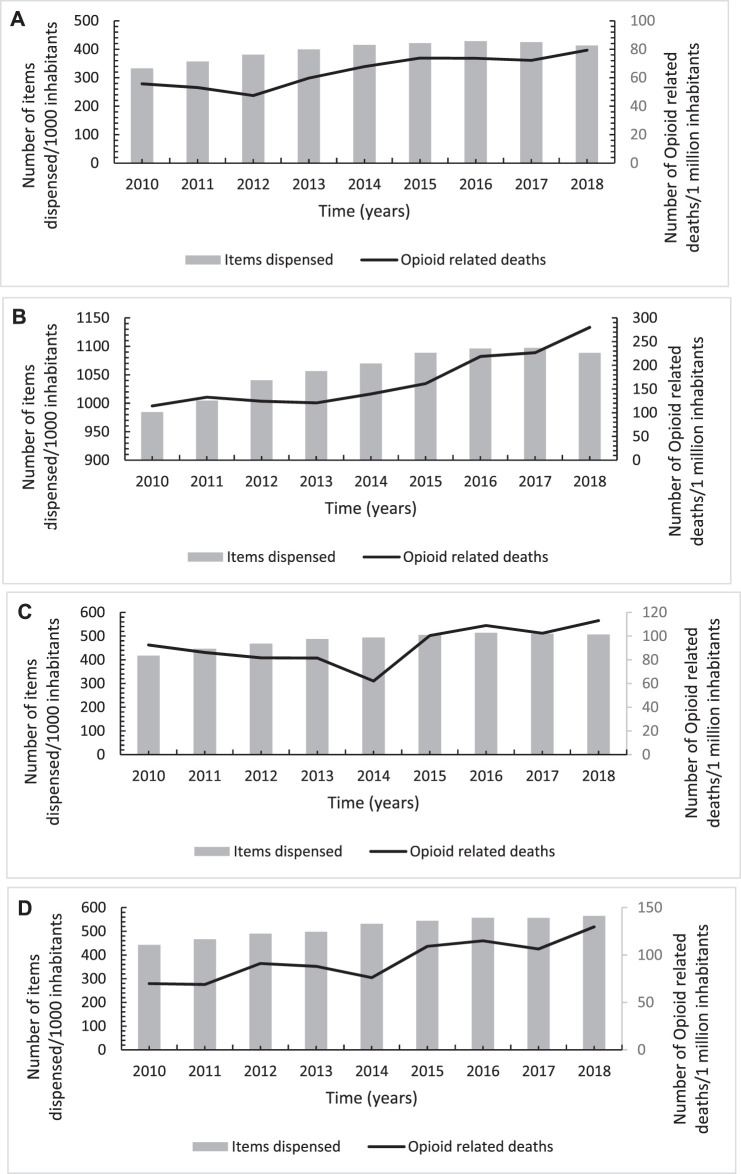
Annual trends in number of opioids-related deaths per one million inhabitants in association with its utilisation trends across England **(A)**, Scotland **(B)**, Wales **(C)** and Northern Ireland **(D)** over the study period from 2010 to 2018.

**FIGURE 7:**
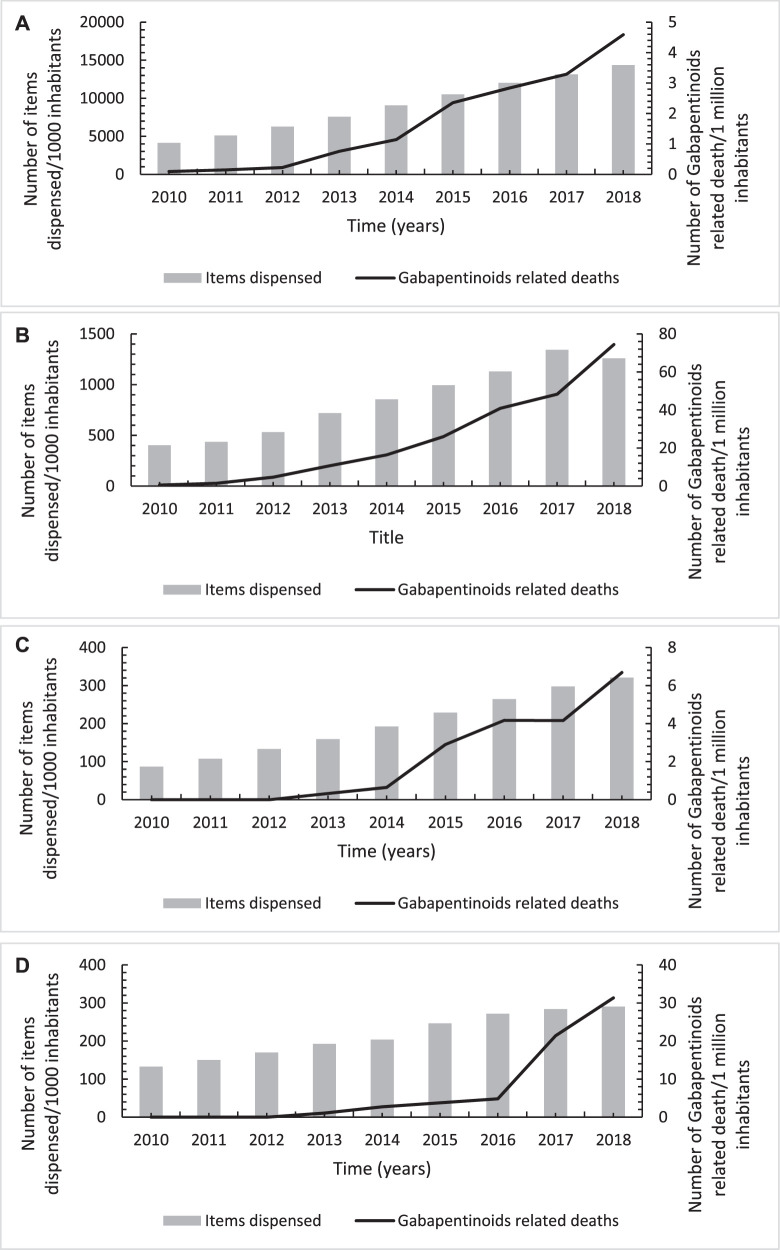
Annual trends in number of gabapentinoids-related deaths per one million inhabitants in association with its utilisation trends across England **(A)**, Scotland **(B)**, Wales **(C)** and Northern Ireland **(D)** over the study period from 2010 to 2018.

The results from the Pearson correlation test indicated a significantly moderate-strong positive correlation between each of opioid and gabapentinoids utilisation and their corresponding related mortality across all the four United Kingdom countries apart from Wales. In Wales, a weak positive correlation between opioid use and opioid-related mortality, albeit non-significant (Figures 6, 7; Table 3).

## Discussion

### Key Findings

We believe this is the first study of its nature assessing and comparing utilisation and mortality-related trends of opioids and gabapentinoids across the four United Kingdom countries. The study results illustrated an overall significant increasing trend in the utilisation of opioids (12.5–14%) and gabapentinoids (205–207%) overtime in the United Kingdom with substantial variations among the four United Kingdom countries. Although the increasing trend for opioid use levelled off and started to decline from 2016 onward, the increasing trends for gabapentinoids use continued over the study period but at a lower rate from 2016 onward compared to the period before 2016. However, this observed levelling off and reducing trends for opioids and gabapentinoids after 2016, respectively, have not being translated into similar declining trends in their related mortality which needs further investigations.

### Strength and Limitations

This is the first cross-national comparative study to asses utilisation trends of opioids and gabapentinoids and their related mortality across the four United Kingdom countries over a 9-year period, using a population-based data that covers the entire United Kingdom population (adults and paediatrics). Furthermore, we have used more than one metric to measure the utilisation trends including DDDs/1,000 inhabitant/day which is a well-recognised international standard utilisation metric for cross-national comparison studies, although we acknowledge the limitation of using DDD for paediatric and young patients in that the observed utilisation trends might be an under or over estimation of the actual trends; however, when there is lack of information on paediatric age or parameters such as indications or prescribed dose (as it was the case in our study), the WHO recommends using DDD as the standard measuring tool in paediatrics with the caveats associated with using an adult based DDD (World Health Organization Collaborating Centre for Drug Statistics and Methodology, 2020). Furthermore, there are some other limitations that need to be acknowledged as well. It was not possible to include codeine products dispensed as an over the counter treatment and any opioids or gabapentinoids dispensed in hospital as our datasets included only prescribed medications in primary care setting. However, there is typically limited dispensing of medicines in out-patients among hospitals in the United Kingdom. Consequently, whilst it is likely our results are an underestimation of the actual utilisation of these medications, we do not believe this has appreciably impacted on our findings. Due to the lack of information on indications, it was not possible to distinguish the actual reason for prescribing these medications; i.e., to treat cancer pain vs. non-cancer chronic pain; hence it was not possible to explain the exact reasons for the observed increasing trends. Consequently, our findings should be interpreted with caution until further research is undertaken regarding the rationale behind our findings. Another major limitation is the fact that our data were aggregated and hence patient-level information on potential confounders were not available (thus was not possible to segment the analysis by these patient-level factors such as age, sex, etc); however, information on potential confounders would have been critical in determining independent risk factors for opioids and gabapentinoids prescribing which was not the focus of our current study; nevertheless, we are planning to use patient level data to investigate the latter in future studies. Furthermore, as our study aimed to assess the strength of association between opioids and gabapentinoids utilisation and their related mortality, we used Pearson correlation coefficient (the standard test for measuring correlations) (Sedgwick, 2012) rather than regression, which is used to quantify the nature of association as a mean of predicting the outcome variable from the predictor variable (Sedgwick, 2013), as it was not the focus of our study due the lack of information on other potential confounders (predictors) affecting opioids and gabapentinoids related mortality due to the ecological, aggregated nature of the datasets used; therefore, our study findings do not imply causal inference/causality and caution is needed to not interpret our study findings prematurely as that increased opioids and gabapentinoids use caused increased opioids and gabapentinoids related mortality.

### Utilisation Trends of Opioids and Gabapentinoids Across the Four United Kingdom Countries

There were significant increasing trends of opioids and gabapentinoids across the four the United Kingdom countries but interestingly with substantial variations among the countries. In terms of number of items dispensed for opioids, Scotland had the highest overall utilisation level, almost three times higher, compared to the lowest observed level in England, followed by NI which had the highest annual increasing rate. NI had not only the second highest utilisation level for total opioids but also the highest utilisation level for strong opioids which is in contrast to Scotland, which despite showing the highest utilisation level for total opioids, it had the highest utilisation level for weak opioids. In terms of utilisation measured by DDDs/1,000 inhabitants/day, we observed similar substantial variations among the four United Kingdom countries but with different utilisation trends when compared with number of items dispensed. NI had the highest utilisation level and annual increasing rate indicating that NI not only utilised high level of strong opioids but also at higher doses which is concerning.

Furthermore, although Scotland’s use of opioids, in terms of number of items dispensed, were mostly for weak opioids, almost two-third of its use in terms of DDDs were for strong opioids suggesting that Scotland appears to prescribe these strong opioids at higher doses. A higher DDDs could be due to either an increased dose, a longer supplied quantity or both but it was not possible to confirm which of these were the reason behind the observed high number of DDDs due to unavailability of dosing frequency in the PCA datasets used in this study. Having said this, a longer supplied quantity is unlikely to be the driving reason as it is a common practice in the United Kingdom to provide one-month supply of medicines especially for Controlled Drugs, including opioids, for which the Department of Health in the United Kingdom has issued strong recommendation that the maximum prescribed quantity should not exceed 30 days (Faculty of Pain Medicine of the Royal College of Anaesthetists, 2017). Furthermore, the issue of prescribing strong opioids at higher doses in the United Kingdom has also been reported by a previous study (Zin et al., 2014).

Due to the lack of previous studies that have evaluated and compared opioid utilisation trends across the four United Kingdom countries, it was not possible to compare out study results with other studies. However, our study findings are consistent and comparable to other studies and reports that have evaluated opioid utilisation trends among individual United Kingdom countries. Several previous studies also reported significant increasing trends of opioids utilisation overtime in the United Kingdom, but these studies were either limited only to England (Mordecai et al., 2018; Chen et al., 2019; Curtis et al., 2019), Scotland (Torrance et al., 2018), Wales (Davies et al., 2019), NI (O’Boyle, 2019) and/or out-dated by including data up to 2014/2015 (Mordecai et al., 2018; Torrance et al., 2018; Chen et al., 2019; Davies et al., 2019).

Similarly, there was significant increasing trends in the utilisation of gabapentinoids across the four the United Kingdom countries over the study period with substantial variations among them ranging from 126.5% in NI to 285.8% in Wales in terms of number of items dispensed which was comparable to the observed figures in terms of number of DDD (i.e., 114.9% in NI to 299.6% in Wales). These findings are consistent and comparable with previous studies/reports which reported significant increasing trends of gabapentinoids in the United Kingdom, even though these were limited to England (The National Institute for Health and Care Excellence, 2019) and Scotland (Smith et al., 2018) with limited published studies on Wales and NI. For instance, there was a 393 and 900% increase in gabapentin and pregabalin prescribing, respectively, over 2007–2017 in England (The National Institute for Health and Care Excellence, 2019). These reported increase rates are greater than what we observed in our current study which is likely attributed to the difference in the study periods in both studies. This observed significant increase in the utilisation trends of gabapentinoids could be attributed to multiple factors including: firstly, their use as alternative to opioids (driven by the pressure from opioids epidemic crisis) as physicians are desperate for safer alternatives to opioids. This resulted in physicians lowering the threshold to prescribe gabapentinoids to treat various types of pain (Goodman and Brett, 2017). Secondly, their frequent use as an off-label to treat non-neuropathic pain, with some clinicians seeing gabapentinoids being used to treat almost any kind of pain (Goodman and Brett, 2017; Montastruc et al., 2018), enhanced by the aggressive pharmaceutical marketing activities (Steinman et al., 2006; Goodman and Brett, 2017). In fact, in the United Kingdom, the number of new patients treated with gabapentinoids has tripled between 2007 and 2017 with 55 and 52% of new pregabalin and gabapentin prescriptions for off-label use (Montastruc et al., 2018). This is of particular concern given the poor- and low-quality evidence of gabapentinoids’ benefit to treat chronic low back pain in comparison with other analgesics (Shanthanna et al., 2017) coupled with their strong potential for dependence, abuse and misuse as a recreational drug (Zaccara et al., 2011). Furthermore, they increase the risk of opioid-related deaths when co-prescribed with opioids (Gomes et al., 2017; Gomes et al., 2018). This is of particular concern in the United Kingdom since gabapentinoids’ co-prescribing has tripled between 2007 and 2017 with a reported co-prescribing rate of 20–25% in 2017 (Montastruc et al., 2018).

Despite the observed overall increasing trends in the use of opioids’ and gabapentinoids’, and consistently with another study (Curtis et al., 2019), it is encouraging that the increasing trends levelled off and were at lower rate for opioids’ and gabapentinoids’, respectively. This could possibly be due to the impact of the multiple strategies/initiatives that have been implemented across the individual United Kingdom countries to address and the tackle the overuse of these medications; these country-specific policies could have also impacted the observed variations in opioids’ and gabapentinoids utilisation among the four United Kingdom countries. These strategies/initiatives included re-classification of tramadol and gabapentinoids (all United Kingdom) (Advisory Council on the Misuse of Drugs, 2013; UK Government, 2018), developing national therapeutic indicators for opioids’ and gabapentinoids’ to optimise their use (England, Wales, Scotland) (NHS Scotland, 2015; The National Institute for Health and Care Excellence, 2017), publishing several educational resources around management and use of opioids’ and gabapentinoids’ in different types of pain such as clinical knowledge summaries and guidelines (England, Wales, Scotland) (The National Institute for Health and Care Excellence, 2013; The National Institute for Health and Care Excellence, 2016a; The National Institute for Health and Care Excellence, 2016b; The Scottish Government and NHS Scotland, 2018) and opioid risk tool (Scotland), publishing information leaflets highlighting the serious risk of addiction associated with opioids (all United Kingdom) (Medicines and Healthcare products Regulatory Agency, 2020), “Opioids aware” in 2016 (all United Kingdom) (Faculty of Pain Medicine of the Royal College of Anaesthetists, 2017) (a website containing information on the clinical use of opioids for pain aiming to support clinicians and patients making fully informed decision on whether to use opioids or not), and involving pharmacists (via community pharmacies and Independent prescriber pharmacist-led pain clinics) to review patients on chronic pain medications, including opioids and gabapentinoids’ (Scotland) (Smith et al., 2018; Hill et al., 2019). However, it is concerning that this observed levelling off of opioids’ and gabapentinoids’ trends from 2016 onward has not been translated into a comparable or similar reduction in the trends of opioids’ and gabapentinoids’-related mortalities. Furthermore, there were strong significant positive correlation between opioids’ and gabapentinoids utilisation trends and their related mortality trends (which we will plan to explore further in future research using patient-level data), that is consistent with findings from other studies conducted in North America (Zerzan et al., 2006; Fischer et al., 2013) and a recent United Kingdom study (Chen et al., 2021) which identified persistence opioid use as a significant independent risk factor for opioid-related mortality. The mechanism of how persistence opioid use might be associated with opioid-death could be explained by the fact that persistence opioid use is often linked to higher risks of adverse effects such as addiction, dependence, abuse, problematic opioid use include co-use with other psychotropics such as benzodiazepines, gabapentinoids and anti-depressants (Ballantyne and LaForge, 2007; Manchikanti et al., 2012; Häuser et al., 2014; Faculty of Pain Medicine of the Royal College of Anaesthetists, 2017), all of which might increase the risk of opioid overdose and/or over response leading subsequently to opioid related-death, especially the co-prescribing with other psychotropics which has been associated with a significant increase in opioid-related death ranging from 2-folds increase for tricyclic antidepressants to 6.2 folds increase for gabapentinoids (Chen et al., 2021).

### Variations in the Utilisation Trends of Opioids and Gabapentinoids Across the Four United Kingdom Countries

Although previous studies have reported regional variations in the use of opioids’ and gabapentinoids within individual United Kingdom country (Mordecai et al., 2018; The Scottish Government and NHS Scotland, 2018; Torrance et al., 2018; Chen et al., 2019; Curtis et al., 2019), our study for the first time illustrated substantial variations in the use of opioids’ and gabapentinoids and their related mortality across the four United Kingdom countries. Several factors have been reported to influence prescribing patterns, including opioids’ and gabapentinoids prescribing; hence introducing regional variations. These factors include 1) population demographics including the proportion of elderly patients (>65 years old), female gender, smokers, obesity and socioeconomic status, with higher utilisation also observed in poorer deprived areas (Mordecai et al., 2018; Chen et al., 2019; Curtis et al., 2019), 2) prevalence of pain-related comorbidities such as depression (Painter et al., 2013; Chen et al., 2019), 3) healthcare providers’ clinical knowledge (Reames et al., 2014) driven primarily by the complex decision-making process in opioids prescribing and pain management (Toye et al., 2017), 4) geography in terms of rural vs. urban populations (Curtis et al., 2019), 5) accessibility, availability and quality of appropriate services in relation to pain management including multidisciplinary services to manage chronic pain (McDonald et al., 2012; Curtis et al., 2019), and 6) local guidelines, formularies and extent of uptake/implementation of opioids/gabapentinoids’-related strategies/initiatives (Reames et al., 2014; Croker et al., 2019). In particular, lower socioeconomic status and deprivation have been considered one of the major determinants for the regional variation in opioids utilisation as these factors have been associated with more chronic pain conditions, persistent opioids use, and aberrant medication-taken behaviour (Day et al., 2006; Mindell, 2012). This is of particular importance and relevance in the United Kingdom given the wide variation in the socioeconomic status among the individual United Kingdom countries (Abel et al., 2016). For example, NI is the most deprived country within the United Kingdom (Abel et al., 2016) and it is where we observed the highest utilisation of strong opioids and gabapentinoids. Issues of healthcare inequality and regional variations in the provision and access of healthcare in the United Kingdom have been also identified in several other disease areas (Lawlor et al., 2003; Williams and Drinkwater, 2009; Martin et al., 2019), which can likely explain some of the observation variations among the United Kingdom countries.

### Implications for Policy Makers and Clinical Practice

Our study findings have important implications for policy makers and clinical practice. The observed significant variations in opioids and gabapentinoids utilisation and their related mortalities indicates that the United Kingdom government and individual United Kingdom countries need to take immediate actions and policies to address this rising issue, in particular NI and Scotland where the issue is the greatest. Scotland had the highest opioids and gabapentinoids related mortality which is of particular concern and needs urgent action. This reaffirms and support the recent demands for radical changes in official United Kingdom policies on drug use after new records show Scotland as having the highest number of drug related deaths not only in the United Kingdom but also in Europe and possibly the world (Christie, 2019), which is consistent with our study findings.

Although the observed increase in opioids and gabapentinoids utilisation might represent better pain management in patients with acute and palliative pain and hence improved quality of life, the prolonged prescribing periods that have been reported elsewhere (Faculty of Pain Medicine, 2017) suggests that this increase is more likely due to unnecessary, inappropriate and dangerous prescribing to treat chronic pain. Furthermore, it is possible that some of implemented strategies/measures in the United Kingdom regarding opioids and gabapentinoids use might have unintentionally resulted in lower use of opioids and gabapentinoids among patients with cancer where rationale use of these medication is considered appropriate to optimise their quality of life (Erskine and Wanklyn, 2021). For example, fear of addiction, promoted through government information leaflet (Medicines and Healthcare products Regulatory Agency, 2020) and adding warnings to the labelling and packaging of opioids about serious risk of addiction, especially for long-term use, might have led to increased reluctance among cancer patients to accept opioids to control their pain (Erskine and Wanklyn, 2021). This could partly explain why the observed levelling off of opioids and gabapentinoids utilisation trends from 2016 onward has not be translated into a comparable or similar reduction in the trends of opioids’ and gabapentinoids’-related mortalities as these reductions could have been among patients who could benefit from long term use of these medications such as those with cancer while unnecessary and unwarranted use kept continuing. However, further research of patient level data coupled with qualitative research is needed before we can say anything with certainty. Our findings, thus, support the calls not only to strike the right balance in prescribing these medications (Erskine and Wanklyn, 2021) but also the need to take effective actions to promote best clinical practice in terms of using these medications at lower doses, for shorter durations and stopping them if they are not beneficial (Foy et al., 2016). One potential way forward is to have effective implementation strategies linked to the currently available guidelines (The National Institute for Health and Care Excellence, 2013; The National Institute for Health and Care Excellence, 2016a; The National Institute for Health and Care Excellence, 2016b; The Scottish Government and NHS Scotland, 2018) to ensure maximum uptake and implementation since it is evident that passive dissemination of guidelines alone without linkage to effective implementation strategies is often associated with failure and guidelines/policy ineffectiveness in the United Kingdom (Baker et al., 2015). This contrasts with changes in prescribing behaviour among physicians in the United Kingdom following multiple interventions (Godman et al., 2018; Godman et al., 2019). Furthermore, there is a need for the United Kingdom government to invest in specialised services as alternative non-pharmacological treatments for pain such as physiotherapy, pain clinics and psychology (Mahase, 2020) especially in deprived areas where the issue of misuse is greatest.

## Conclusion

The utilisation trends of opioids and gabapentinoids have increased significantly over the study period in the four United Kingdom countries with substantial variations among countries with the highest level in Scotland and NI. However, although it is encouraging that the trends started to level off from 2016 onward, it is concerning that the latter was not translated into a comparable and similar decline in opioids and gabapentinoids-related mortalities with Scotland having the highest mortality rate. Our findings support the call for immediate actions to address this rising issue including radical changes in official United Kingdom policies on drug use as well as effective strategies to improve and promote best clinical practice in opioids and gabapentinoids prescribing.

## Data Availability

The original contributions presented in the study are included in the article/supplementary material, further inquiries can be directed to the corresponding author.
